# Effect of Electrosynthesis Potential on Nucleation, Growth, Adhesion, and Electronic Properties of Polypyrrole Thin Films on Fluorine-Doped Tin Oxide (FTO)

**DOI:** 10.3390/polym13152419

**Published:** 2021-07-23

**Authors:** Jhon Puerres, Pablo Ortiz, María T. Cortés

**Affiliations:** 1 Chemistry Department, Universidad de los Andes, Bogotá D.C. 111711, Colombia; jd.puerres@uniandes.edu.co; 2 Chemical Engineering Department, Universidad de los Andes, Bogotá D.C. 111711, Colombia; portiz@uniandes.edu.co

**Keywords:** electrosynthesis, polypyrrole, thin films, nucleation and growth, adhesion, electronic properties

## Abstract

Polypyrrole (PPy) is one of the most attractive conducting polymers for thin film applications due to its good electrical conductivity, stability, optical properties, and biocompatibility. Among the technologies in which PPy has gained prominence are optoelectronics and solar energy conversion, where transparent electrodes such as fluorine-doped tin oxide (FTO) or indium tin oxide (ITO) are frequently used. However, FTO substrates have the notable advantage that their components are widely available in nature, unlike those of ITO. Recognizing the importance that the FTO/polypyrrole system has gained in various applications, here, we studied for the first time the nucleation and growth mechanism of electro-synthesized PPy on FTO. Additionally, the effect of the synthesis potential (0.9, 1.0, 1.1, and 1.2 V vs. Ag/AgCl) on the homogeneity, adhesion, conductivity, and HOMO energy levels of PPy films was determined. From current–time transients and scanning electron microscopy, it was found that films synthesized at 0.9 and 1.0 V exhibit 3D growth with progressive nucleation (as well as lower homogeneity and higher adhesion to FTO). In contrast, films synthesized at 1.1 and 1.2 V follow 2D growth with instantaneous nucleation. It was also evident that increasing the polymerization potential leads to polymers with lower conductivity and more negative HOMO levels (versus vacuum). These findings are relevant to encourage the use of electro-synthesized PPy in thin film applications that require a high control of material properties.

## 1. Introduction

Since the discovery of intrinsically conductive polymers (ICPs) by Shirakawa et al. in the 1970s [1], significant efforts have been made to understand the properties of these materials that combine the characteristics of metals and plastics. Over time, potential applications have been investigated in several areas where ICPs perform as functional materials, for example, batteries [2,3,4], solar cells [5,6,7], electrochromic devices [8,9,10], sensors [11,12,13], and more recently in photocatalysis and photoelectrochemistry [14,15,16,17,18]. Commonly, the performance of ICPs is strongly related to their morphology, redox state, and structural order (chain arrangement and conjugation length) as these parameters influence the surface area, electronic, and optical properties of the polymers [19,20]. An important feature of these materials is that these properties can be conveniently tuned from the synthesis conditions, for example, by changing the dopant nature [21], the oxidizing agent [22], introducing additives to form copolymers [23], modifying the synthesis solvent [24], adjusting the pH of the precursor solution [25], etc. Precisely, electrochemical polymerization is known as a very versatile technique to obtain ICPs because it is the one that best allows fine tuning of polymer properties [26], with the advantage that coatings are obtained directly on conductive and semiconductive substrates [27]. In this sense, the understanding of the electro-polymerization process (nucleation and growth mechanism) is necessary for the use of these polymers in thin film applications, which require homogeneous coatings with high thickness control and appropriate electronic properties.

The nucleation and growth of conducting polymers have typically been investigated by current–time transients or microscopic and spectroscopic measurements. From these types of studies, it has been accepted that the nucleation and growth mechanism of ICPs is similar to that of metals, so the theoretical curves of nucleation and growth derived by Harrison and Thirsk have been frequently used to analyze the electrosynthesis of these polymers [28,29]. This model considers instantaneous and progressive nucleation, as well 2D and 3D growth, and it can be expressed in terms of dimensionless quantities for potentiostatic polymerizations as shown in Equations (1)–(4). *I* and *t* represent current and time, respectively, and *I_m_* and *t_m_* represent the coordinates at the maximum current [30,31]. In instantaneous nucleation, the number of nuclei remains constant during polymerization, and growth occurs over the initial positions without the formation of new nuclei. Consequently, in this nucleation, the radii of the nuclei tend to be large, and the surface morphology is rough. In the case of progressive nucleation, there is a continuous formation of new nuclei in the course of polymerization. Thus, growth occurs on all types of nuclei, recent and initially formed, leading to a surface morphology with a tendency to be flatter. In 3D growth, the rate of nuclei growth is practically equal in the parallel and perpendicular directions with respect to the electrode surface, whereas in 2D growth, nuclei extend faster laterally (in the direction parallel to the electrode) until they meet and overlap [29,32].

Three-dimensional (3D) growth and instantaneous nucleation:

(1) $${\left(\frac{I}{I_{m}}\right)}^{2}=\frac{1.9542{\left\{1-exp\left[-1.2564\left(t/t_{m}\right)\right]\right\}}^{2}}{\left(\frac{t}{t_{m}}\right)}.$$

Three-dimensional (3D) growth and progressive nucleation:

(2) $${\left(\frac{I}{I_{m}}\right)}^{2}=\frac{1.2254{\left\{1-exp\left[-2.3367{\left(t/t_{m}\right)}^{2}\right]\right\}}^{2}}{\left(\frac{t}{t_{m}}\right)}.$$

Two-dimensional (2D) growth and instantaneous nucleation:

(3) $$\left(\frac{I}{I_{m}}\right)=\left(\frac{t}{t_{m}}\right)exp\left\{\frac{-1}{2}\left[{\left(\frac{t}{t_{m}}\right)}^{2}-1\right]\right\}.$$

Two-dimensional (2D) growth and progressive nucleation:

(4) $$\left(\frac{I}{I_{m}}\right)={\left(\frac{t}{t_{m}}\right)}^{2}exp\left\{\frac{-2}{3}\left[{\left(\frac{t}{t_{m}}\right)}^{3}-1\right]\right\}.$$

Studies on the electrosynthesis of conducting polymers have shown that the nucleation and growth mechanism of these materials depends on variables such as the type of monomer, the monomer concentration, the applied potential, the electrode material, etc. For example, Dian et al. found that the polymerization of selenophene, 3-methylselenophene, and 3-ethylselenophene follows a 3D growth with progressive nucleation, while the polymerization of 3,4-dimethylselenophene presents a 3D growth with instantaneous nucleation (in all cases, using ClO_4_^−^ as dopant and platinum as electrode) [33]. Mandić et al. reported that the type of nucleation presented in the electrosynthesis on platinum of polyaniline (PANI) doped with ClO_4_^−^ changes with the monomer concentration from 3D growth with progressive nucleation (0.1 M aniline) to 3D growth with instantaneous nucleation (0.15 M aniline) [34]. Shustak et al. observed that pyrrole polymerization on stainless steel follows a 3D growth with instantaneous nucleation, but if stainless steel is modified with n-alkanoic acid monolayers, the polymerization of pyrrole takes a 3D growth with progressive nucleation (in both cases using tetrafluoroborate as dopant and applying 1.6 V or 1.7 V vs. Ag/AgBr). When they applied lower potentials, the authors found no differences in the nucleation and growth mechanism of polypyrrole on both substrates [32].

On the other hand, some authors have reported changes in the nucleation and growth mechanism during polymerization and/or a combination of two mechanisms at the same stage (two mechanisms occurring in parallel). For example, in the study of Hwang et al. it was found that the polymerization of pyrrole on highly oriented, gold-coated pyrolytic graphite follows a 2D growth with instantaneous nucleation before nuclei overlapping and a 3D growth with progressive nucleation after nuclei overlapping. However, the authors observed that a heat treatment of the substrates (250 °C for 10 min) before polymerization led to 3D growth with progressive nucleation throughout the electrosynthesis due to a decrease in surface defects [29]. In other study, Hwang et al. synthesized PANI on highly oriented pyrolytic graphite using SO_4_^2−^ as dopant. In this polymerization, a first stage (before nuclei overlapping) corresponded to a 2D growth with instantaneous nucleation, a second stage at maximum current (during nuclei overlapping) was associated with a 3D growth with progressive nucleation, and a last stage (after nuclei overlapping) was related to a combination between a 2D growth with instantaneous nucleation and a 3D growth with progressive nucleation [30].

Regarding the electronic properties of ICPs, the HOMO (highest occupied molecular orbital) and LUMO (lowest unoccupied molecular orbital) energy levels are especially important for the fabrication of organic electronics [35]. The separation between HOMO and LUMO defines the band gap of the material (E_g_), while the position of the energy levels versus the vacuum determines the potential of the polymer for some applications. For instance, the HOMO level is a useful criterion for the design of photoelectrodes [36] and components in organic and hybrid solar cells [37,38].

The determination of HOMO and LUMO levels in ICPs is usually performed by cyclic voltammetry (using 0.1 M NBu_4_PF_6_ in acetonitrile as electrolyte), which is a dynamic electrochemical technique that allows characterizing the redox (reduction and oxidation) properties of materials. Equations correlating the electrochemical oxidation and reduction onset potentials of ICPs with their corresponding HOMO and LUMO energies are shown below.

(5) $$E^{\mathrm{HOMO}}=-\left(E_{onset\;ox.}+5.1\right)\;\left(\mathrm{eV}\right)$$

(6) $$E^{\mathrm{LUMO}}=-\left(E_{onset\;red.}+5.1\right)\;\left(\mathrm{eV}\right)$$

Here, *E_onset ox._* and *E_onset red._* are the onset potentials of oxidation and reduction, respectively, vs. the ferrocene (Fc)/ferrocenium (Fc^+^) redox couple, and 5.1 eV is a correction factor corresponding to the energy of the Fc/Fc^+^ pair on the Fermi scale [35,39].

Among ICPs, polypyrrole is recognized for its very favorable characteristics, such as high electrical conductivity, optical properties, chemical stability, and biocompatibility [40]. Therefore, in recent years, this polymer has gained relevance in thin film applications, including hydrogen production [41], energy storage [42], and solar energy conversion [43], where transparent conductive electrodes are frequently used. Among these electrodes, indium tin oxide (ITO) has been broadly used owing to its high transmittance and low electrical resistance. However, as indium is a limited natural resource on Earth, fluorine-doped tin oxide (FTO) has become an attractive alternative because of the notable advantage that its components are widely available in nature [44,45,46].

Considering that polypyrrole has a functional role in various thin film applications, it is very fruitful to tune its properties from the electrosynthesis conditions. In this work, the nucleation and growth mechanism of polypyrrole on FTO substrates was studied by chronoamperometry experiments. Furthermore, the effect of the polymerization potential on the adhesion, homogeneity, conductivity, and HOMO levels of the polymer was determined. Different mechanisms were found to predominate as a function of the applied potential, which in turn influenced the adhesion and homogeneity of the films.

## 2. Materials and Methods

### 2.1. Materials

The 25 mm × 12.5 mm FTO substrates (Ossila, TEC 8, Sheffield, UK) were initially cleaned for 3 min using a 10% *w*/*w* NaOH (Carlo Erba, ≥97%, Val de Reuil, France) at 55 °C; subsequently, the substrates were subjected to two sonications in ultrapure water during 15 min each and using an operating frequency of 42 kHz. Pyrrole (sigma Aldrich, 98%, Steinheim am Albuch, Germany) was distilled under a nitrogen atmosphere using a heat gun (GHG 180 Bosch, Gerlingen, Germany) with an air flow of 450 L/min at 170 °C. Acetonitrile (Sigma Aldrich, HPLC grade, Darmstadt, Germany) was stored over molecular sieves. LiClO_4_ (Sigma Aldrich, ≥95% St. Louis, MI, USA) and NBu_4_PF_6_ (Sigma Aldrich, ≥99.0%, St. Louis, MI, USA) were used without further purification.

### 2.2. Electropolymerization Process

Pyrrole polymerizations were carried out at room temperature (~14 °C) in a conventional three-electrode cell using a platinum foil as counter electrode and an Ag/AgCl (3M NaCl) electrode (MF-2052, BASi, West Lafayette, IN, USA) as reference electrode (RE). The synthesis solutions (composed of 0.25 M pyrrole and 0.5 M LiClO_4_ in acetonitrile +2% *w*/*w* H_2_O) were deoxygenated with nitrogen for 10 min before each experiment. Electrochemical polymerizations were carried out on freshly cleaned FTO substrates under potentiostatic conditions at 0.9, 1.0, 1.1, and 1.2 V vs. RE. The amount of deposited polymer was controlled by the supplied electric charge. In this system, 0.9 V was very close to the minimum potential to achieve pyrrole oxidation on FTO (Figure S1). The potentiostat used in all experiments was an Autolab PGSTAT302N (Utrecht, The Netherlands).

### 2.3. Characterizations

The morphology of the PPy films was examined by scanning electron microscopy (SEM) using a TESCAN LYRA3 equipment (Brno–Kohoutovice, Czech Republic) at an accelerating voltage of 10.0 kV. The RMS roughness of the FTO was measured by atomic force microscopy using a tapping mode with an Asylum Research MFP-3D-BIO equipment (Goleta, CA, USA). The adhesion of the polymer films to the FTO substrates was evaluated in triplicate by a cross-cut tape adhesion test. For this purpose, cross-sections with inter-slit gaps of approximately 1 mm were made on each PPy film. Then, the tape (ASTM D 3359-B, Elcometer, Manchester, UK) was pressed onto the film and stripped off. UV-Vis spectra were measured with an Analytik Jena SPECORD 50 PLUS spectrophotometer (Jena, Germany). HOMO energy levels of the polymer films were determined in triplicate by cyclic voltammetry in a solution (0.1 M NBu_4_PF_6_ in acetonitrile) deoxygenated with nitrogen for 10 min before each test. Here, platinum was used as a counter electrode and an Ag/AgCl electrode was used as RE. The sheet resistance and conductivity of the polymer films were measured in triplicate with an Ossila T2001A2 four-point probe system (Sheffield, UK) using a target current of 100 μA and a voltage increment of 0.05 V.

## 3. Results and Discussion

### 3.1. Electrosynthesis of Polypyrrole on FTO

It is known that pyrrole dissolved in an appropriate solvent also containing an electrolyte is oxidized at the interface of a working electrode by applying an anodic potential. This process involves a sequence of reactions in which each coupling step has to be activated by two species [47] (detailed discussions about these coupling steps are found in the literature [47,48,49,50]). Regarding the electrochemical stoichiometry, for each mol of reacting monomer, 2.25 to 2.33 moles of electrons are consumed. Here, two moles of electrons go to polymerization, while the additional electrons are consumed in the oxidation of the polymer and its consequent doping [48]. In this way, the complete reaction can be expressed as [47]:

(7) $$\left(n+2\right)\mathrm{HPyH}⟶\mathrm{HPy}{\left(\mathrm{Py}\right)}_{n}{\mathrm{PyH}}^{\left(\mathrm{nx}\right)+}+\;\left(2n+2\right)H^{+}+\;\left(2n+2+\mathrm{nx}\right){\;e}^{-}$$

where HPyH is the monomer (pyrrole), and the expression (2n + 2 + nx) e^−^ can be separated in (2n + 2) electrons for polymerization and nx electrons for doping. Now, it is important to mention that the reactivity of the monomeric species is high, but in oligomeric species, the reactivity decreases drastically. For this reason, in a first step, a radical ion dimerization of monomeric molecules occurs, but the coupling tendency between charged oligomers and a monomeric radical cation decreases as a function of the length of the oligomeric chain [47]. This kinetic limitation can be addressed to some extent by adjusting the electrosynthesis potential, as it has been observed that increasing the oxidation potential results in longer polymer chains [47,49].

**Figure 1:**
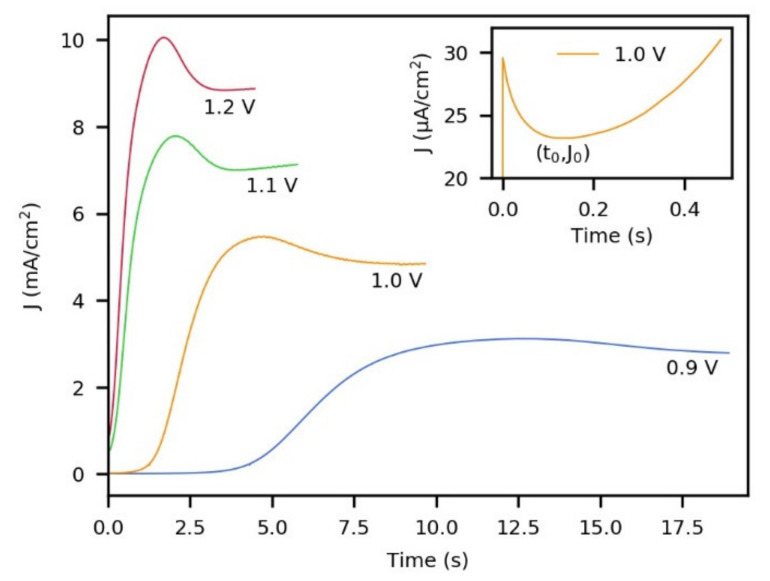
Current–time transients for the electropolymerization of pyrrole on FTO substrates at different anodic potentials. Synthesis solution (0.25 M pyrrole and 0.5 M LiClO_4_ in acetonitrile +2% *w*/*w* H_2_O). The inset shows a magnification of the curve at 1.0 V at a very early time in the electrosynthesis.

Typical current–time transients obtained in this work during the electrochemical polymerization of pyrrole at different anodic potentials are shown in Figure 1. Here, each curve is shown until an electric charge of 37.6 mC/cm^2^ is reached. It is clear that the rate of polymer deposition changes drastically with oxidation potential, which is reflected in different times and current densities required to reach the set electric charge. In all the cases, the transients presented a maximum current, and when the applied potential was gradually increased from 0.9 to 1.2 V, the value of the maximum current increased and was reached in a shorter time. This maximum current has been associated with the spread and collapse (overlapping) of polymeric nuclei regardless of the substrate used [30,40,51] and also with the existence of a diffusion-controlled process [52]. The inset in Figure 1 shows a magnification of the curve at 1.0 V in the first 0.5 s of the electrosynthesis. This early stage is known as induction or incubation time and is characterized by a current peak followed by a subsequent decay of the current to a minimum with coordinates: t_0_, J_0_. This region has been attributed to double layer charging and monomer oxidation, and it is commonly missed in nucleation and growth studies since nuclei formation begins after t_0_ [33,53].

**Figure 2:**
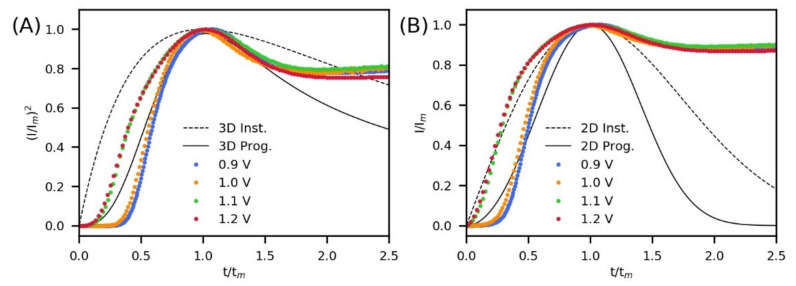
Dimensionless plots of maximum currents (Figure 1) and theoretical curves for instantaneous and for progressive nucleation with (**A**) 3D growth and (**B**) 2D growth during the electrosynthesis of polypyrrole.

The comparison between the experimental transients and theoretical curves was performed using the parameters t_m_* and J_m_* shown in Table 1, which were calculated from t_m_* = *t_m_* − *t*_0_ and J_m_* = *J_m_* − *J*_0_ (correction by the induction stage). The dimensionless curves obtained are shown in Figure 2A,B for instantaneous/progressive 3D growth and instantaneous/progressive 2D growth, respectively. It is observed that before reaching the maximum current, the nucleation and growth mechanism for PPy is affected by the potential. Thus, two types of behavior are observed, one for 0.9 V and 1.0 V and the other for 1.1 V and 1.2 V. After the maximum current, the nucleation and growth mechanism of PPy on the FTO appears to be the same for all the potentials used in this study.

From the comparation with the 3D theoretical transients (Figure 2A), it is observed that the electrosynthesis curves obtained at 0.9 V and 1.0 V follow a 3D growth with progressive nucleation around the maximum current (in a range that can be stablished as *t*/*t_m_* > 0.65 and *t*/*t_m_* < 1.5). However, noticeable deviations are found during the early stage of polymerization and also during the thickening of the films. In the case of the electrosynthesis curves obtained at 1.1 V and 1.2 V, there was not a good match with any theoretical 3D curve before the maximum current and also not after *t*/*t_m_* > 1.5. Comparing the experimental data with the 2D theoretical transients (Figure 2B), some correspondence is observed between the instantaneous nucleation (2D) and the curves obtained at 1.1 V and 1.2 V before the current maximum. For a better comparison with the mechanisms, the fits of the theoretical curves with the experimental data were evaluated by the coefficient of determination R^2^ (Table 2) [32]. In addition, the standard error (S) was calculated for each model and synthesis potential to provide a measure of how far (on average) the experimental values are from theoretical points. In all cases, R^2^ and S values were determined to analyze the models between *t*/*t_m_* > 0 and *t*/*t_m_* < 1.5, since after *t*/*t_m_* > 1.5, the transients at all potentials were significantly away from the theoretical ones. The results show that the highest R^2^ values for 0.9 V and 1.0 V were obtained with the 3D growth with progressive nucleation model, and for 1.1 V and 1.2 V, they were obtained with the 2D growth model with instantaneous nucleation. These results agree with the calculated standard errors, since the lowest S value corresponds to the highest R^2^ for each polymerization potential.

**Figure 3:**
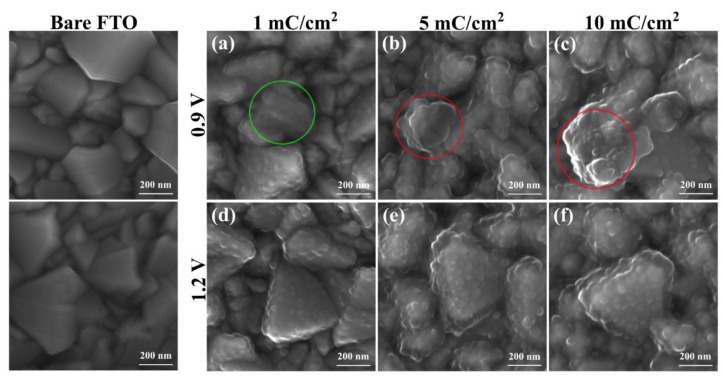
SEM images of PPy synthesized on FTO with electric charge control (mC/cm^2^). (**a**–**c**): polymerization at 0.9 V vs. Ag/AgCl; (**d**–**f**): polymerization at 1.2 V vs. Ag/AgCl.

Some discrepancies between experimental and theoretical data could be mainly attributed to deviations from Faraday’s law (which explains the electrodeposition of metals) due to the formation of oligomers as previously reported [40,54]. Therefore, SEM micrographs of the electropolymerized PPy at 0.9 V and 1.2 V and under electric charge control were taken to obtain further evidence of the nucleation and growth mechanism (Figure 3). The bare substrate image showed FTO grains with a wide variety of sizes and shapes; this was consistent with the high RMS roughness (25.26 nm) measured by atomic force microscopy (Figure S2). At potentials of 0.9 V and 1.2 V, at a very early stage of polymerization (1 mC/cm^2^), the deposition of small PPy nuclei on the FTO grains was appreciated (Figure 3a,d). At 0.9 V, these nuclei were only present on a few grains, giving rise to bare areas of the electrode (green circle). In contrast, for the synthesis at 1.2 V, the nuclei were noted to be homogeneously distributed over the FTO grains (Figure 3d). It was also observed that at this stage, the diameters of the nuclei were generally less than 50 nm and were clearly spaced, indicating the absence of overlap at both potentials. At electric charges of 5 mC/cm^2^ and 10 mC/cm^2^, PPy accumulations were observed in some areas (red circles) when synthesized at 0.9 V (Figure 3b,c). As for PPy synthesized at 1.2 V, it showed a growth trend following the shape of FTO grains (Figure 3e,f). This is in agreement with 3D growth at 0.9 V and 1.0 V and 2D growth at 1.1 V and 1.2 V, as similar results were obtained at 1.0 V and 1.1 V (Figure S3).

Here, it is worth mentioning that our results show that the nucleation and growth mechanism of polypyrrole on FTO may differ from that reported on ITO. For example, Castro-Beltran et al. reported 3D growth with progressive nucleation for Cl^−^-doped PPy on ITO-coated polyester, in this case evaluating only one synthesis potential (0.8 V vs. Ag/AgCl) [40]. On the other hand, Longo et al. found 3D growth with instantaneous nucleation for ClO_4_^−^-doped PPy on ITO glass, here also working with only one synthesis potential (0.8 V vs. SCE) [26]. In any case, a strict comparation of the mechanisms is difficult due to the few studies that exist on the topic and the great variability in the synthesis conditions that can be used.

### 3.2. Adhesion and Homogeneity of Polypyrrole Films on FTO

**Figure 4:**
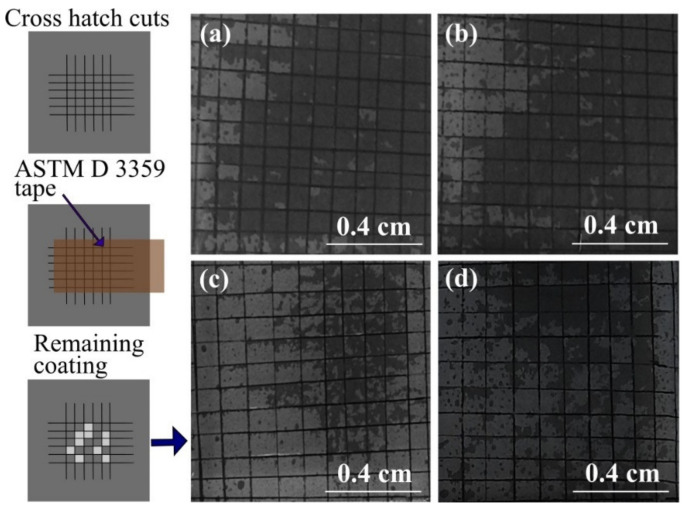
ASTM D 3359 tape adhesion test for PPy films on FTO substrates. The photos show the coatings remaining on the substrate after peeling off the tape. The films were synthesized at different potentials: (**a**) 0.9 V, (**b**) 1.0 V, (**c**) 1.1 V, and (**d**) 1.2 V. The electric charge supplied was 21 mC/cm^2^ in all cases.

The adhesion of polypyrrole on different electrode surfaces is known to be poor due to the lack of strong molecular interactions between PPy and electrodes, which usually limits the practical applications of these coatings [55]. Herein, the adhesion of polypyrrole to FTO substrates was evaluated by a cross-cut tape adhesion test (Figure 4) to assess the effect of the polymerization potential. In this way, after performing the adhesion test in triplicate, the mean value of the remaining coverage of the polymer on the substrate was determined, 73% ± 1% (0.9 V), 71% ± 3% (1.0 V), 27% ± 6% (1.1 V), and 25% ± 5% (1.2 V). These results show that the best adhesions are obtained at potentials of 0.9 V and 1.0 V, while 1.1 V and 1.2 V lead to very low adhesions of PPy to FTO. Considering the differences found in the nucleation and growth mechanism of the polymer on the FTO, the best adhesions (at 0.9 V and 1.0 V) could be attributed to the fact that a 3D growth with progressive nucleation results in globular shape of various sizes, which may imply a higher number of anchoring points to the substrate. This is different for 2D growth with instantaneous nucleation, since in this case, the initially formed nuclei tend to follow the shape of the substrates without the formation of new nuclei (which could act as new anchor points).

**Figure 5:**
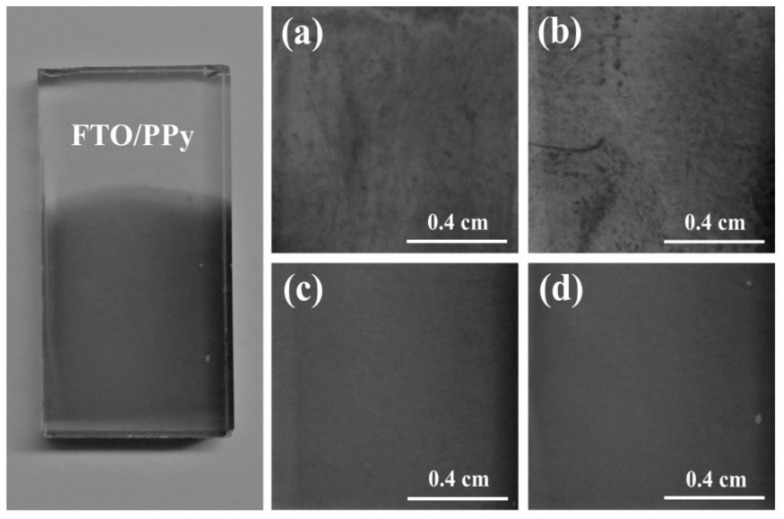
Photos of polypyrrole films synthesized at: (**a**) 0.9 V, (**b**) 1.0 V, (**c**) 1.1 V, and (**d**) 1.2 V (vs. Ag/AgCl). Synthesis solution (0.25 M pyrrole and 0.5 M LiClO_4_ in acetonitrile +2% *w*/*w* H_2_O). In all cases, the electric charge supplied was 10 mC/cm^2^. The complete photo shows PPy on FTO synthesized at 1.2 V.

On the other hand, as can be seen in Figure 5, important differences were found in the homogeneity of the PPy films as a function of the synthesis potential when the same electric charge of polymerization was used (10 mC/cm^2^). PPy synthesized at 0.9 V and 1.0 V are not homogeneous and show some areas darker than others, indicating polymer accumulation. In contrast, PPy synthesized at 1.1 V and 1.2 V show a total and homogeneous coverage of the surface. These results could be expected from differences in the nucleation and growth mechanism of PPy films. A 2D growth implies that the nuclei spread faster in the parallel direction to the electrode surface, so it could be predicted that a polymer with this kind of growth reaches full substrate coverage using a lower electric charge compared to the same polymer following a 3D growth. In this case, complete coverage of the FTO substrates was achieved using electric charges higher than 20 mC/cm^2^ regardless of the synthesis potential.

### 3.3. UV-Vis Absorption and Electronic Properties of Polypyrrole Films Electro-Synthesized on FTO

**Figure 6:**
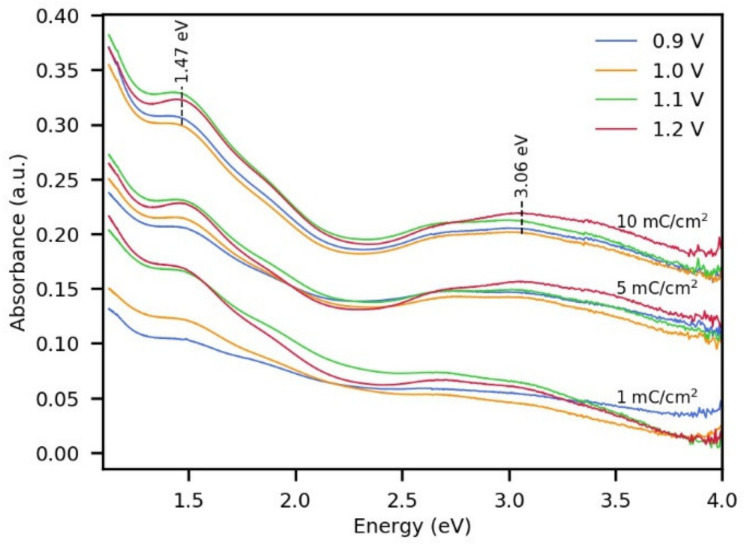
UV-Vis spectra of PPy deposited on FTO under control of electric charge and potential during the synthesis.

UV-Vis spectra were taken to analyze the absorbance properties of the deposited PPy at different potentials (Figure 6). An increase in absorption was observed at all wavelengths as the electric charge of synthesis was higher, which is consistent with the increase in the amount of polymer deposited. Two absorption bands at 3.06 eV and 1.47 eV were identified in the spectra, which are characteristic of polypyrrole in the oxidized state [56,57]. The signal at 3.06 eV is associated with the π–π* transition of PPy, and the absorption at 1.47 eV is assigned to the presence of charge carriers (bipolarons) in PPy [58] (as a consequence of the doping process during electrosynthesis). It is highlighted that the PPy obtained with an electric charge as low as 1 mC/cm^2^ presented a broad absorption in the NIR region, suggesting that even before nuclei overlapping (Figure 3), the electrosynthesis involves both the oxidation of pyrrole for polymer growth and the oxidation of the formed PPy chains. Regarding the effect of the synthesis potential on the absorption spectra of PPy, no differences were observed using 5 and 10 mC/cm^2^. However, using 1 mC/cm^2^, a lower absorption in the visible and NIR region was appreciated when PPy was obtained at 0.9 V and 1.0 V. This may point to the fact that at this very early stage of the synthesis, PPy has a lower degree of oxidation when obtained at 0.9 V and 1.0 V compared to when obtained at 1.1 V and 1.2 V.

**Figure 7:**
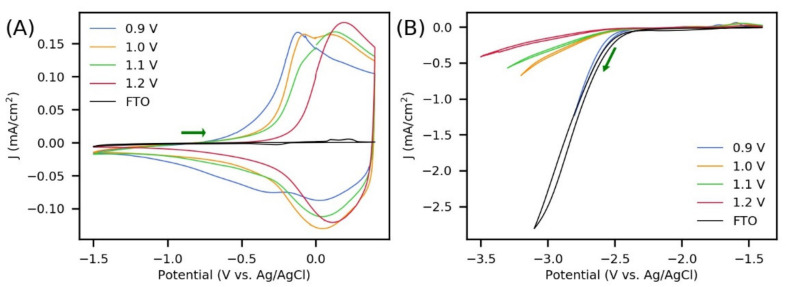
Cyclic voltammograms of polypyrrole films synthesized at 0.9 V, 1.0 V, 1.1 V, and 1.2 V on FTO substrates. Potential window for (**A**) oxidation and (**B**) reduction of PPy. In all cases, the electric charge supplied during polymerization was 42 mC/cm^2^. Electrolyte: 0.1 M NBu_4_PF_6_ in acetonitrile, scan rate of 20 mV/s.

The redox properties of PPy films were characterized in triplicate by cyclic voltammetry to determine changes in HOMO and LUMO energy levels with the polymerization potential. Two potential windows were chosen in order to observe separately the change from neutral to oxidized PPy (Figure 7A) and the change from neutral to reduced PPy (Figure 7B). The required conversion from the Ag/AgCl reference to the Fc/Fc^+^ reference to make use of Equations (5) and (6) was performed by subtracting 0.44 V from the potentials versus Ag/AgCl, since 0.44 V corresponds to the half-wave potential of Fc/Fc^+^ at the FTO/PPy electrodes (Figure S4).

Regarding the oxidation of PPy synthesized with 42 mC/cm^2^ (Figure 7A), cyclic voltammograms showed a shift of the oxidation signal toward less negative potentials as the polymerization potential increased. Furthermore, all films showed reversible behavior, as can be seen from the well-defined currents in the reverse potential scans. This redox activity is associated with doping/de-doping (charge/discharge) processes, which involve the exchange of ions and solvent molecules between the polymer and the electrolyte solution to ensure charge and osmotic balance [50]. The oxidation onset potentials were determined as the intercept of the tangents of the baseline and the slope of the oxidation peak (see Figure S5). Table 3 shows the average HOMO energy level values and their relative standard deviations (number of samples *n* = 3). Here, a variation of 0.27 eV is observed between the PPy synthesized at 0.9 V and that obtained at 1.2 V (for polymerization charges of 42 mC/m^2^). The same measurements were carried out for PPy synthesized with 21 mC/cm^2^ (Figure S6), and a similar shift of the oxidation signal according to the polymerization potential was observed (Table 3). However, all HOMO levels were less negative than those obtained for PPy synthesized with 42 mC/cm^2^, and in this case, a variation of up to 0.29 eV was noticed. An important advantage of the electrochemical synthesis is its high reproducibility, which is reflected in the low relative standard deviations in HOMO energy level measurements (Table 3). In Figure S7, cyclic voltammograms of three different samples of PPy obtained at 1.2 V (42 mC/cm^2^) are shown.

Significant differences in the HOMO and LUMO levels of PPy are found in the literature: for example, HOMO levels vs. vacuum between −5.46 and −6.21 eV and LUMO levels vs. vacuum between −3.61 and −4.19 eV [59,60,61]. Even so, there are few reports about strategies for tuning energy levels of PPy. In particular, some examples on this aspect have considered the formation of composite materials [61,62], the insertion of substituents into the monomer [63], and the insertion of new dopants into the polymer [64]. However, as far as we know, there are no reports on variations in the HOMO level of PPy when adjusting the electrosynthesis potential. These observed variations could be attributed to differences in the following factors: bond length alternation energy (E^BLA^), aromatic resonance energy (E^res^), torsional angle energy (E^θ^), and intermolecular interactions energy (E^int^), which determine the energy difference between the HOMO and LUMO levels in ICPs (E_g_) [65,66,67]. In the case of E^BLA^, it is associated with the difference between single and double bond lengths. While the aromatic form in ICPs is energetically more stable, the quinoid form has a higher energy and leads to lower E_g_. E^res^ is related to the energy difference between the aromatic structure and a hypothetical structure with localized single and double bonds. Lower E^res^ leads to lower E_g_. E^θ^ is associated to the torsional angle (θ) between adjacent aromatic units. The flatter the conjugated backbone of the polymer, the lower the E_g_. Lastly, E^int^ is determined by the intermolecular interactions between the backbones: the greater the interaction between the polymer chains, the lower the E_g_ [65,66,67].

Otherwise, when performing cyclic voltammetry to analyze the reduction of PPy synthesized with 42 mC/cm^2^ (Figure 7B), a strong electrochemical response of the bare FTO was observed in that potential window. This signal could be related to the reduction of Sn^4+^ (present in the FTO) to a lower valence state or to elemental form [68]. This strong substrate signal and the insertion of the solvent inside the films made it difficult to determine the reduction onset of the polymer. For this reason, it was not possible to obtain reliable LUMO level values with the two polymerization charges used in this work. This was different to the observed in the determination of the HOMO levels, where no interference from the oxidation signals of the FTO substrates was appreciated (Figure 7A).

Sheet resistance and conductivity measurements of the polymer films were performed in triplicate. Since these characterizations require that the films are not supported on a conductive material (such as FTO), a high electric charge (420 mC/cm^2^) was supplied during polymerization to easily separate the films from the substrates. To detach the films from the FTO electrodes, a tape was pressed onto each film and carefully peeled off to avoid causing cracks in the polymer. Since the thickness of PPy is directly related to the electric charge supplied, a thickness of 0.87 µm was calculated (for 420 mC/cm^2^) following a previous report [69] (see Supplementary Materials). The calculated thickness was used in the software (Ossila Sheet Resistance, version 2.0.4.0, Sheffield, UK) supplied in the four-point probe system to obtain the conductivity measurements. The results of the characterizations and their respective relative standard deviations are shown in Table 4. It is clear that increasing the polymerization potential resulted in films with higher sheet resistances and thus lower conductivities. Specifically, an approximately sevenfold decrease in the conductivity of PPy was found when the electrosynthesis potential changed from 0.9 to 1.2 V. This can be related to an increase in cross-linked networks during polymerization, as high oxidation potentials are known to lead not only to longer chain lengths but also to structural defects due to the generation of highly charged and reactive intermediates [47]. Differences in the polymeric structure as a function of the polymerization potential may be one of the main causes of the shift in the oxidation signal noted in Figure 7A and Figure S6. Although in HOMO level measurements, the electric charge supplied was 21 mC/cm^2^ and 42 mC/cm^2^ (corresponding to approximately 43 nm and 87 nm, respectively [69]), it has been observed that cross-linking is a parallel process to the polymerization and polymer oxidation during the electrochemical synthesis of ICPs [50,70].

Considering the results of this work, it is evident that some properties of PPy electro-synthesized on FTO are strongly related to the polymerization potential. However, it is known that several electrosynthesis conditions affect the properties of PPy on this type of transparent conductive substrates. For example, Alizadeh et al. reported the effect of dopant on the optoelectronic properties of electro-synthesized PPy on FTO [71]. Meanwhile, for PPy electro-deposited on ITO, it has been observed that dopant modifications leads to different degrees of adhesions and energy levels [40,64]. Regarding temperature, it was found (using ITO electrodes) that the conductivity of PPy decreased by two orders of magnitude when the temperature was adjusted from 2 to 75 °C [48]. It has also been reported that lowering the temperature can increase the synthesis yield as a result of the decrease in the solubility of the oligomers, this despite a lower reaction rate [47]. In this way, for further studies about the electrosynthesis of PPy on FTO, it would be interesting to address other conditions, including solvent type, temperature, and monomer and electrolyte concentrations.

## 4. Conclusions

The electrochemical synthesis of polypyrrole on FTO electrodes was carried out using perchlorate as dopant. The mechanism of polymer nucleation and growth at different constant potentials was explored by comparing current–time transients with theoretical curves. The results were contrasted with observations by scanning electron microscopy and complemented with adhesion measurements and determination of conductivity and HOMO energy levels of the polymeric films. It was found that the polypyrrole films obtained at 0.9 V and 1.0 V vs. Ag/AgCl have a progressive nucleation and a 3D growth on FTO, while those synthesized at 1.1 V and 1.2 V vs. Ag/AgCl follow an instantaneous nucleation with a 2D growth. By the cross-cut tape adhesion test, average values of remanent coating on the substrate of 73%, 71%, 27%, and 25% were found for PPy synthesized at 0.9 V, 1.0 V, 1.1 V, and 1.2 V, respectively, suggesting a relationship between the nucleation and growth mechanism and the adhesion of the polymer to FTO substrates. On the other hand, with the increase of the polymerization potential, it was observed that HOMO levels (vs. vacuum) of PPy tend to be more negative and that the conductivity of the films tends to decrease. These findings are relevant to encourage the use of electrochemically synthesized polypyrrole in applications that require high control of material properties.

## Figures and Tables

**Table 1:** Parameters of the electropolymerization of pyrrole at different anodic potentials obtained from the curves in Figure 1.

Parameter	0.9 V	1.0 V	1.1 V	1.2 V
J_m_* (mA/cm^2^)	3.107	5.451	7.257	9.220
t_m_* (s)	11.92	4.47	2.00	1.67
*t*_0_ (s)	0.74	0.15	0.02	0.01
*J*_0_ (µA/cm^2^)	11	23	528	832

**Table 2:** Coefficients of determination (R^2^) and standard errors (S) calculated by fitting the experimental data with the theoretical models (Equations (1)–(4)).

Potential (V)	3D Inst. (R2)	3D Prog. (R2)	2D Inst. (R2)	2D Prog. (R2)	3D Inst. (S)	3D Prog. (S)	2D Inst. (S)	2D Prog. (S)
0.9	0.36	0.97	0.82	0.87	0.32	0.07	0.17	0.14
1.0	0.32	0.96	0.81	0.87	0.34	0.08	0.17	0.14
1.1	0.72	0.92	0.97	0.43	0.19	0.10	0.06	0.24
1.2	0.73	0.90	0.97	0.34	0.18	0.11	0.05	0.24

**Table 3:** HOMO energy levels of PPy films synthesized at different potentials and under control of electric charge (n = 3). These values were calculated according to Equation (5).

Polymerization Potential (V)	Polymerization Charge (mC/m^2^)	HOMO (eV)	Relative Standard Deviation (%)
0.9	42	−4.26	0.9
1.0	42	−4.36	0.9
1.1	42	−4.42	0.9
1.2	42	−4.53	0.9
0.9	21	−4.14	2
1.0	21	−4.26	1
1.1	21	−4.32	1
1.2	21	−4.43	2

**Table 4:** Sheet resistance and conductivity of PPy films synthesized at different potentials and under control of electric charge (420 mC/cm^2^).

Polymerization Potential (V)	Sheet Resistance (Ω/sq)	Relative Standard Deviation (%)	Conductivity (S/m)	Relative Standard Deviation (%)
0.9	90	2	1.27 × 10^4^	2
1.0	143	1	8.0 × 10^3^	1
1.1	2.9 × 10^2^	3	4.0 × 10^3^	2
1.2	6.6 × 10^2^	3	1.7 × 10^3^	4

## Data Availability

Data is contained within the article and the Supplementary Materials.

## Supplementary Materials

The following are available online at https://www.mdpi.com/article/10.3390/polym13152419/s1. Figure S1. Cyclic voltammograms for the potentiodynamic synthesis of PPy-ClO_4_ on FTO electrodes. Synthesis solution: 0.25 M Pyrrole + 0.5 M LiClO_4_ in acetonitrile +2% *w*/*w* H_2_O. Scan rate of 20 mV/s. The red cycle denotes the first polymerization cycle; Figure S2. AFM characterization of bare FTO; Figure S3. SEM images of PPy on FTO deposited controlling the electric charge supplied. (**a**–**c**): polymerization at 1.0 V vs. Ag/AgCl; (**d**–**f**): polymerization at 1.1 V vs. Ag/AgCl; Figure S4. Cyclic voltammogram of ferrocene/ferrocenium on FTO/PPY. Electrolyte: 2.7 mM ferrocene + 0.1 M NBu_4_PF_6_ in acetonitrile, scan rate of 20 mV/s; Figure S5. Cyclic voltammograms of the oxidation of polypyrrole synthesized at: (**A**) 0.9 V, (**B**) 1.0 V, (**C**) 1.1 V, and (**D**) 1.2 V vs. Ag/AgCl. In all cases, the electric charge supplied during polymerization was 42 mC/cm^2^. Electrolyte: 0.1 M NBu_4_PF_6_ in acetonitrile, scan rate of 20 mV/s; Figure S6. Cyclic voltammograms of the oxidation of polypyrrole synthesized at: (**A**) 0.9 V, (**B**) 1.0 V, (**C**) 1.1 V, and (**D**) 1.2 V vs. Ag/AgCl. In all cases, the electric charge supplied during polymerization was 21 mC/cm^2^. Electrolyte: 0.1 M NBu_4_PF_6_ in acetonitrile, scan rate of 20 mV/s; Figure S7. Cyclic voltammograms of polypyrrole films (three samples) synthesized at 1.2 V vs. Ag/AgCl. For all polymerizations, the electric charge supplied was 42 mC/cm^2^. Electrolyte: 0.1 M NBu_4_PF_6_ in acetonitrile, scan rate of 20 mV/s. Reference [69] is cited in the Supplementary Materials.
