# Supplementary material for: Effect of Electrosynthesis Potential on Nucleation, Growth, Adhesion, and Electronic Properties of Polypyrrole Thin Films on Fluorine-Doped Tin Oxide (FTO)
Source: Polymers (Basel). 2021 Jul 23;13(15):2419. doi: 10.3390/polym13152419 (PMC8347362; doi:10.3390/polym13152419)
Supplement: Supplementary file 1 [file polymers-13-02419-s001.zip › polymers-1276176-supplementary.pdf]

polymers

Supplementary Materials

Effect of Electrosynthesis Potential on Nucleation, Growth, Adhesion, and Electronic Properties of Polypyrrole Thin Films on Fluorine-Doped Tin Oxide (FTO)

Jhon Puerres [1], Pablo Ortiz [2] and María T. Cortés [1,*]

[1] Chemistry Department, Universidad de los Andes, Bogotá D.C. 111711, Colombia; jd.puerres@uniandes.edu.co
[2] Chemical Engineering Department, Universidad de los Andes, Bogotá D.C. 111711, Colombia; portiz@uniandes.edu.co
* Correspondence: marcorte@uniandes.edu.co; Tel.: +57-1-3394949-3132

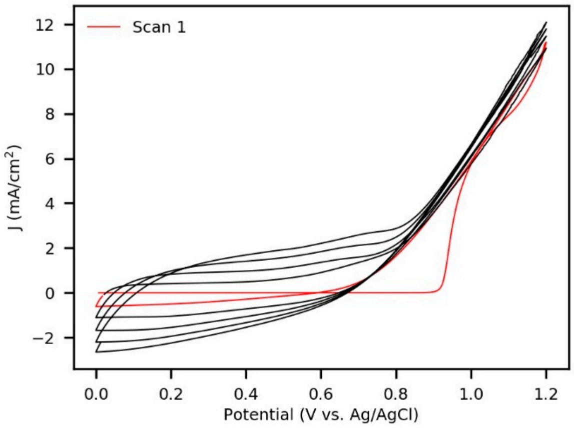

Figure S1. Cyclic voltammograms for the potentiodynamic synthesis of PPy-ClO_4 on FTO electrodes. Synthesis solution: 0.25 M Pyrrole + 0.5 M $LiClO_4$ in acetonitrile + 2% w/w H_2O. Scan rate of 20 mV/s. The red cycle denotes the first polymerization cycle.

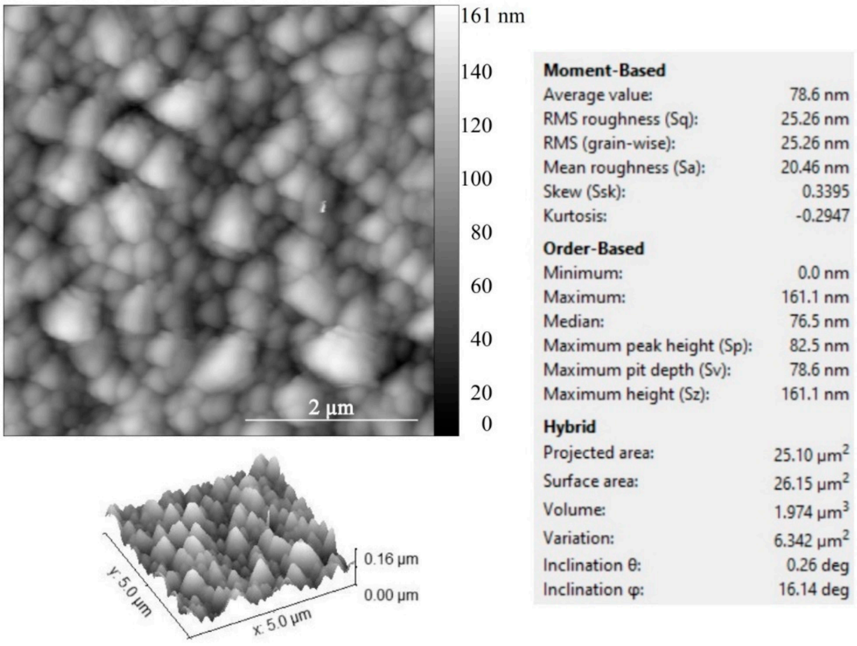

Figure S2. AFM characterization of bare FTO.

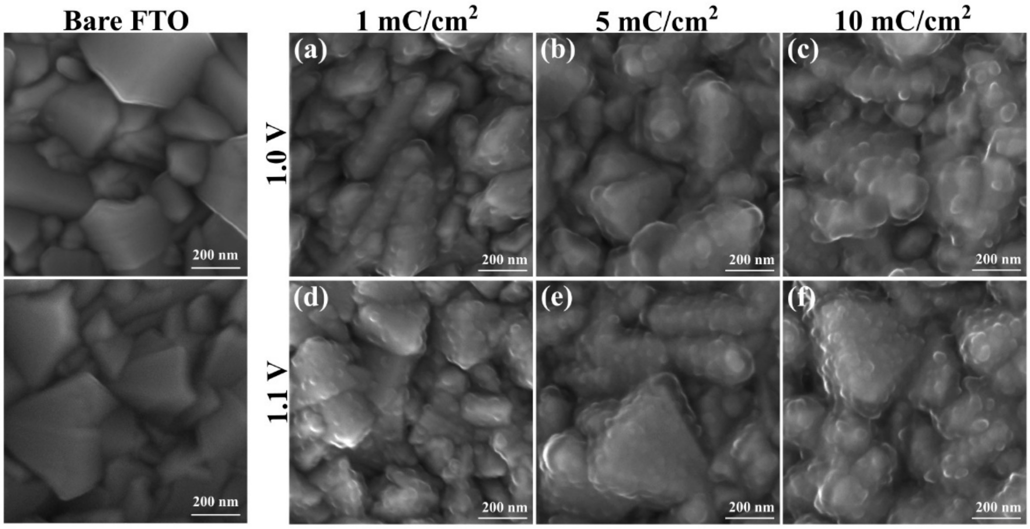

Figure S3. SEM images of PPy on FTO deposited controlling the electric charge supplied. (**a-c**): polymerization at 1.0 V vs. Ag/AgCl; (**d-f**): polymerization at 1.1 V vs. Ag/AgCl.

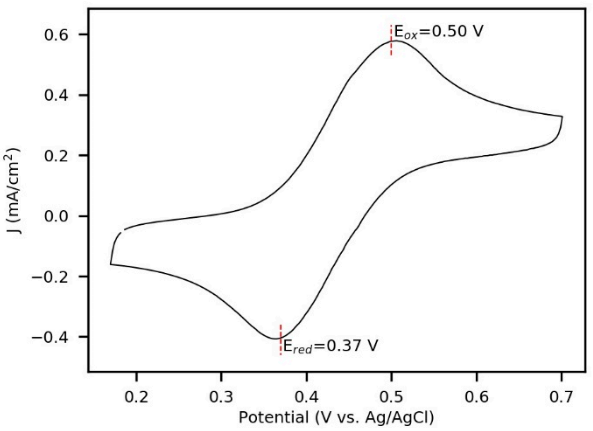

Figure S4. Cyclic voltammogram of ferrocene/ferrocenium on FTO/PPY. Electrolyte: 2.7 mM ferrocene + 0.1 M NBu_4PF_6 in acetonitrile, scan rate of 20 mV/s.

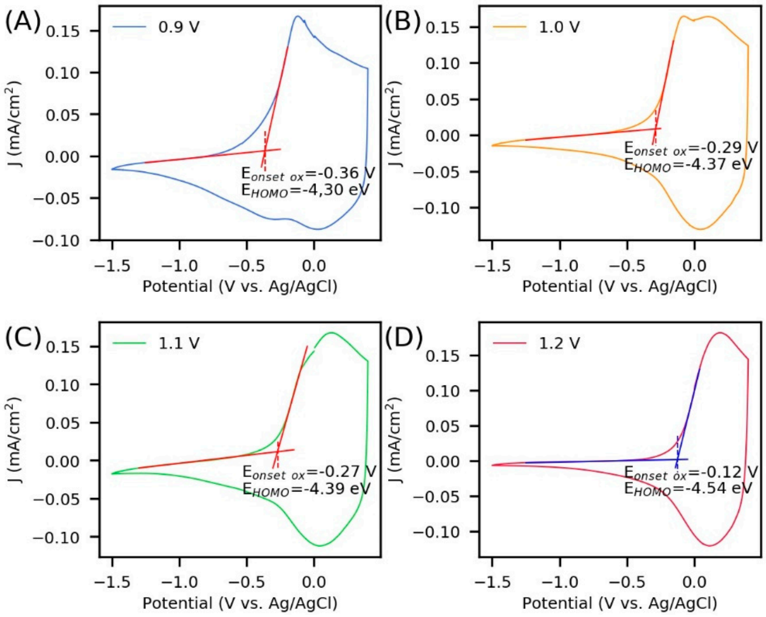

Figure S5. Cyclic voltammograms of the oxidation of polypyrrole synthesized at: (**A**) 0.9 V, (**B**) 1.0 V, (**C**) 1.1 V, and (**D**) 1.2 V vs. Ag/AgCl. In all cases, the electric charge supplied during polymerization was 42 mC/cm^2. Electrolyte: 0.1 M NBu_4PF_6 in acetonitrile, scan rate of 20 mV/s.

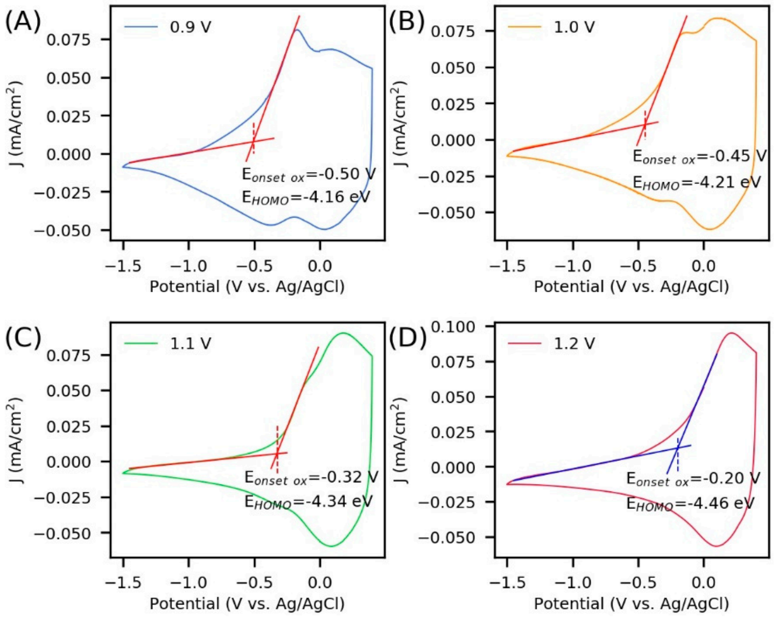

Figure S6. Cyclic voltammograms of the oxidation of polypyrrole synthesized at: (**A**) 0.9 V, (**B**) 1.0 V, (**C**) 1.1 V, and (**D**) 1.2 V vs. Ag/AgCl. In all cases, the electric charge supplied during polymerization was 21 mC/cm^2. Electrolyte: 0.1 M NBu_4PF_6 in acetonitrile, scan rate of 20 mV/s.

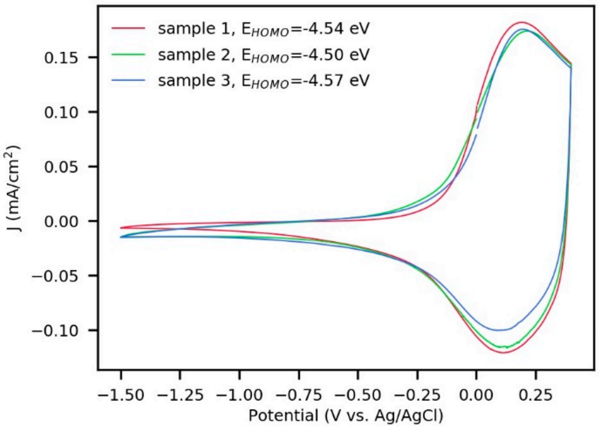

Figure S7. Cyclic voltammograms of polypyrrole films (three samples) synthesized at 1.2 V vs. Ag/AgCl. For all polymerizations, the electric charge supplied was 42 mC/cm2. Electrolyte: 0.1 M NBu4PF6 in acetonitrile, scan rate of 20 mV/s.

Calculation of the thickness of polypyrrole films:

Thickness (cm) = QM/ϱzF, where Q is the electric charge density (C/cm^2), M is the molar mass of pyrrole (67.09 g/mol), ϱ is the polymer density (1.5 g/cm^3), z is the electron loss (2.25) and F is the Faraday constant (96500 C/mol) [69].
